# Wireless Sensor Network-Based Service Provisioning by a Brokering Platform

**DOI:** 10.3390/s17051115

**Published:** 2017-05-12

**Authors:** Luis Guijarro, Vicent Pla, Jose R. Vidal, Maurizio Naldi, Toktam Mahmoodi

**Affiliations:** 1 Institute ITACA, Universitat Politècnica de València, 46022 València, Spain; lguijar@upv.es (L.G.); vpla@upv.es (V.P.); 2 Dipartimento di Ingegneria Civile e Ingegneria Informatica, University of Rome Tor Vergata, 00173 Roma, Italy; maurizio.naldi@uniroma2.it; 3 Center of Telecommunications Research, King’s College London, London WC2R 2LS, UK; toktam.mahmoodi@kcl.ac.uk

**Keywords:** wireless sensor networks, two-sided markets, service provision

## Abstract

This paper proposes a business model for providing services based on the Internet of Things through a platform that intermediates between human users and Wireless Sensor Networks (WSNs). The platform seeks to maximize its profit through posting both the price charged to each user and the price paid to each WSN. A complete analysis of the profit maximization problem is performed in this paper. We show that the service provider maximizes its profit by incentivizing all users and all Wireless Sensor Infrastructure Providers (WSIPs) to join the platform. This is true not only when the number of users is high, but also when it is moderate, provided that the costs that the users bear do not trespass a cost ceiling. This cost ceiling depends on the number of WSIPs, on the value of the intrinsic value of the service and on the externality that the WSIP has on the user utility.

## 1. Introduction

The “Internet of Things” (IoT) is one of the hottest topics being debated today across industries worldwide. The estimate of the number of smart objects in homes, offices, factories, vehicles and elsewhere is 50 billion by 2020, up from 12.5 billion in 2010 [1]. Although smart objects are becoming omnipresent, the fact is that the market for services related to these objects is immature.

The aim of this work is to contribute to the understanding of a sustainable business model for wireless-sensor-network-based services, which is a likely scenario for the IoT. Specifically, this paper proposes a business model built around a brokering platform that distributes the sensing information to the relevant parties and takes care of bundling the solutions, setting the tariffs, billing the customers and providing customer care for the variety of services and applications envisaged for the Internet of Things [1].

In the analysis of this platform-based business model for IoT, we borrow the concept of two-sided markets, as presented by [2] and by [3] and as analysed by [4]. Armstrong [2] defines multi-sided markets as “markets in which two or more groups of agents interact via intermediaries or platforms. Surplus is created—or destroyed in the case of negative externalities—when the groups interact.In a set of interesting cases, cross-group externalities are present, and the benefit enjoyed by a member of one group depends upon how well the platform does in attracting custom from the other group. A brief list of other such markets includes: credit cards (for a given set of charges, a consumer is more likely to use a credit card that is accepted widely by retailers, while a retailer is more likely to accept a card that is carried by more consumers); television channels (where viewers typically prefer to watch a channel with fewer commercials, while an advertiser is prepared to pay more to place a commercial on a channel with more viewers); and shopping malls (where a consumer is more likely to visit a mall with a greater range of retailers, while a retailer is willing to pay more to locate in a mall with a greater number of consumers passing through).” Rochet and Tirole [3] roughly define multi-sided markets as “markets in which one or several platforms enable interactions between end-users and try to get the two (or multiple) sides ‘on board’ by appropriately charging each side [...]. Examples of two-sided markets readily come to mind. Video-game platforms, such as Atari, Nintendo, Sega, Sony Play Station, and Microsoft X-Box, need to attract gamers in order to persuade game developers to design or port games to their platform, and they need games to induce gamers to buy and use their video-game console. Software producers court both users and application developers, client and server sides, or readers and writers. Portals, TV networks, and newspapers compete for advertisers as well as ‘eyeballs.’ And payment card systems need to attract both merchants and cardholders.” However, they go further and define “a two-sided market as one in which the volume of transactions between end-users depends on the structure and not only on the overall level of the fees charged by the platform”.

Some specifics related to the WSN’s operation are incorporated in the model, such as the influence of the sensing rate on the user utility and on the WSN’s cost structure. We investigate how the pricing schemes that a platform applies to each side (users and wireless sensor networks) may increase the total service take-up at each side [5].

As far as the authors are aware, there are some papers that discuss which requirements a sustainable business model should comply with in an IoT scenario ([6,7] and the references therein), but there are only a few papers that approach this issue as formally as our work [8,9,10].

The work in [8] provides a survey of the pricing schemes for IoT services and proposes a business model where the provider intermediates between sensors and users, like in our work. Several providers are modelled, and the goal is to analyse whether providers will cooperate in offering their IoT service as a bundle or not and, if so, how to optimize the bundled subscription fee. The scope is therefore different from our work.

Interestingly, in [9] the authors model the competition in prices in the provision of IoT services. The theoretical framework is information economics, which departs from ours. The model is simple, since the information source is binary, but the approach is novel and promising because it can be applied to model time-sensitive information and information reselling.

In a preliminary study [10], we have studied a business model for WSN-based service provision also built around a brokering platform. The results obtained there were partial, the discussion limited and the description of the mathematical analysis was kept to a minimum. This paper extends our preliminary study so that the analysis conducted and the results presented are exhaustive and complete.

The structure of the paper is as follows. The next section describes the business model. Section 3 provides a detailed presentation of the analysis and the derivation of the results. Section 4 discusses the results of the paper, and Section 5 draws the conclusions.

## 2. Business Model

The scenario modelled in this paper comprises *N* Wireless Sensor Infrastructure Providers (WSIPs), one service provider and *M* users. A monopolistic service provider is assumed. We acknowledge that no barrier can be identified in this market, so that more realistic scenarios where several service providers compete against each other should be modelled. However, at the current stage, the study of a monopolistic model can be regarded as representative and can provide valuable insights to approach the study of more complex scenarios (we analysed a scenario with competing service providers in [11], where we simplified the service provider’s business model to the extent that it does not operate as a platform aimed at creating a two-sided market, but uses a simple linear price scheme in the WSN’s side instead; however, the results obtained in [11] are not comparable to the ones obtained in this paper).

### 2.1. Wireless Sensor Infrastructure Providers

Each WSIP operates and manages a Wireless Sensor Network (WSN). The WSN island senses information that is bundled by the service provider in order to compose useful services for the users.

WSN *j* is able to sense at a rate $r_{j}$. This rate not only influences the user utility, as stated below, but also contributes to the costs incurred. Specifically, we model WSIP *j*’s costs as proportional to its sensing rate, i.e., $f\cdot r_{j}$, modelling the fact that the more a WSN senses, the more resources it consumes, e.g., battery. We model the heterogeneity among the WSNs in terms of $r_{j}$ through a random variable R uniformly distributed in the interval $\left[0,1\right]$.

**Figure 1:**
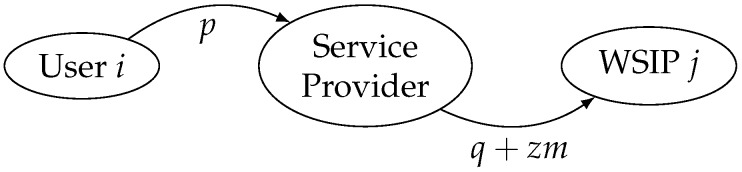
Payment flow.

We assume that the service provider pays a fixed fee plus an amount *z* per subscriber to each connected WSIP, i.e., the total payment is q+zm, where *q* is the fixed fee and *m* is the number of users that subscribe to the service (the proposed payment is different from a two-part tariff. While the proposed payment depends on the opposite side to the side where it is applied, a two-part tariff does not). The payment flow is shown in Figure 1. This payment is intended to create a double incentive for the WSIPs to join the platform, since they will be rewarded collectively as more users subscribe.

Therefore, provided that WSIP *j* joins the service provider platform, it will get the profit:

(1) $$\Pi_{j}=q+zm-fr_{j}.$$

Otherwise, the WSIP will get zero revenue and profit. The number of WSIPs that join the platform is denoted by *n*.

### 2.2. Users

Users are interested in accessing a range of services that the service provider composes from the WSN islands operated by the WSIPs.

Each user has a utility that comprises both objective aspects and unobserved aspects. The unobserved aspects may reflect subjective features of the service consumption, and these aspects are responsible for the heterogeneity of the user consumption behaviour. We propose to model these aspects with a uniform random variable that reduces the objective part of the utility in a linear manner. More specifically, each user has a type denoted by $x_{i}$, which is modelled as a uniformly-distributed random variable $X=U\left[0,1\right]$, and a dis-utility equal to $x_{i}$ multiplied by a cost factor *t* reduces the objective part of the utility (this approach can be also interpreted as a generalization of the Hotelling model, where: $x_{i}$ is the user physical location; the service provider is located at x=0; and $tx_{i}$ is the transportation cost).

As regards the objective aspects, the sensor nodes produce utility by sensing and reporting data to the WSIP and, ultimately, to the service provider. Therefore, following [12], the utility that the users get from the WSN-based service is assumed to depend on the aggregate sensing rate $\sum_{j=1}^{n}r_{j}$, through a positive, non-decreasing and concave function Φ(·).

Provided that user *i* subscribes for the service, his/her utility is then specified by:

(2) $$u_{i}=v+\Phi \left(\sum_{j=1}^{n}r_{j}\right)-tx_{i}-p,$$

where the intrinsic utility *v* is the net value that a user receives from accessing the platform irrespective of the amount of service received, accounting also for network access fees; and *p* is the lump-sum payment for the service. We assume that a user will get zero utility if he/she chooses not to subscribe to the service.

From Equation (2), it follows that there always exists a cross externality from the number of WSIPs to the number of users; that is, the more WSIPs join the platform, the greater utility the users get. This intrinsic cross externality is supplemented by the cross externality created by the proposed payment in (1). A bidirectional cross externality is then generated. This may recreate a two-sided market where the service provider acts as a platform. If so, the service provider would internalize the bidirectional cross externality, improve its profit and increase the take-up of either the users, or the WSIPs, or both.

### 2.3. Service Provider

The service provider performs two basic roles in the model: it composes services that are based on the information sensed by the different WSN islands; and it acts as an intermediary between users and WSIPs, which allows decoupling the pricing schemes on each side.

The profit of the service provider is given by the revenues from the users (pm) minus the cost incurred in paying the WSIPs ((q+zm)n):

(3) $$\Pi_{p}=pm-\left(q+zm\right)n.$$

## 3. Analysis

In this section, the expressions for the number of subscribers and of connected WSIPs are derived, and the profit-maximizing prices set by the service provider are obtained.

Let us assume that the number of WSIPs that join the platform is $n^{e}$ and that the rates from each of these WSIPs are $r_{j}^{e},j=1,\ldots ,n^{e}$. User *i* will subscribe to the service if $u_{i}\geq 0$, which occurs with probability:

(4) $$P\left(u_{i}\geq 0\right)=P\left(X\leq \frac{1}{t}\left(v+\Phi \left(\sum_{j=1}^{n^{e}}r_{j}^{e}\right)-p\right)\right)=\Psi \left(\frac{1}{t}\left(v+\Phi \left(\sum_{j=1}^{n^{e}}r_{j}^{e}\right)-p\right)\right),$$

where Ψ is defined as follows:

(5) $$\Psi \left(u\right)\equiv \left\{\begin{array}{cc} 0 & \mathrm{if}\;u<0\\ u & \mathrm{if}\;0\leq u<1\\ 1 & \mathrm{if}\;1\leq u. \end{array}\right.$$

The number of subscribers M is then a random variable, and its expected value, conditioned to the values $n^{e},r_{1}^{e},r_{2}^{e},\ldots ,r_{n^{e}}^{e}$, is equal to:

(6) $$m\equiv E\left[M|n^{e},r_{1}^{e},r_{2}^{e},\ldots ,r_{n^{e}}^{e}\right]=M\Psi \left(\frac{1}{t}\left(v+\Phi \left(\sum_{j=1}^{n^{e}}r_{j}^{e}\right)-p\right)\right).$$

We proceed now in a similar way for the number of connected WSIPs. Let us assume that the number of subscribers is $m^{e}$. WSIP *j* will join the platform if $\Pi_{j}\geq 0$, which corresponds to a random event with probability:

(7) $$P\left(q+zm^{e}-fR\geq 0\right)=\Psi \left((q+zm^{e})/f\right).$$

Following a similar reasoning as with *m*, the expected number of connected WSIPs is equal to:

(8) $$n\equiv E\left[N\right]=N\Psi \left((q+zm^{e})/f\right).$$

Additionally, the rate R of a connected WSIP has an expected value equal to:

(9) $$r\equiv E\left[R|R\leq (q+zm^{e})/f\right]=\frac{1}{2}\Psi \left((q+zm^{e})/f\right)=n/2N.$$

We look for fulfilled expectations equilibria [13] where each side’s expectations are fulfilled in Equations (6) and (8) (an equivalent assumption is that all agents have a perfect foresight [14]),

(10) $$\begin{array}{cc} m^{e} & =m, \end{array}$$

(11) $$\begin{array}{cc} n^{e} & =n, \end{array}$$

(12) $$\begin{array}{cc} \sum_{j=1}^{n^{e}}r_{j}^{e} & =n\cdot r, \end{array}$$

so that Equation (6) becomes:

(13) $$m=M\Psi \left((v+\Phi (nr)-p)/t\right),$$

and Equation (8) becomes:

(14) $$n=N\Psi \left((q+zm)/f\right).$$

Then, the expressions for *m* and *n* can be obtained by solving the system of Equations (13) and (14).

Assuming that the monopoly platform is free to set both subscription price *p* for the users and fee *q* for the providers, the platform faces the problem of choosing *p* and *q* to maximize its profit $\Pi_{p}$. Thus, a profit maximization problem should be solved, as is described below.

A squared root function has been chosen for Φ, following the examples considered in [12], i.e., $\Phi \left(nr\right)=b\sqrt{nr}=bn/\sqrt{2N}$. Parameter *b* gives the degree of cross externality from the WSIPs to the users.

Since Ψ is a piece-wise linear function defined in three different intervals, solving Equations (13) and (14) yields nine solution types for (m,n): m=n=0; m=0, 0<n<N; m=0, n=N; 0<m<M, n=0; 0<m<M, 0<n<N; 0<m<M, n=M; m=M, n=0; m=M, 0<n<N; and m=M, n=M. Each solution has a feasibility region in the pq-plane that is delimited by a piece-wise linear curve. By substituting the solution into (3), in each region, we have a function $\Pi_{p}\left(p,q\right)$ that is a polynomial of degree one or two. To solve the maximization problem, we first solve a constrained maximization problem in each of the nine regions and then compare the obtained maxima among them to obtain the global maximum.

To simplify notation, we introduce the following definitions:

(15) $$\begin{array}{cc} A & \equiv \frac{Nf}{M} \end{array}$$

(16) $$\begin{array}{cc} B & \equiv \frac{Mb}{f\sqrt{8N}} \end{array}$$

(17) $$\begin{array}{cc} C & \equiv \frac{v+b\sqrt{N/2}}{2} \end{array}$$

(18) $$\begin{array}{cc} D & \equiv \frac{t}{Mz}-\frac{b\sqrt{N/2}}{f}. \end{array}$$

The following lemma will be used several times through this section.

**Lemma** **1.** C=v/2+AB.

### 3.1. Expressions for the Expected Number of Customers (m) and WSIPs (n)

First, we consider the nine solution types for (m,n) arising from Equations (13) and (14). As noted $\Phi \left(nr\right)=\frac{b}{\sqrt{2N}}n$, so that Equations (13) and (14) can be rewritten as:

(19) $$\begin{array}{cc} m & =M\Psi \left(\frac{v+\frac{b}{\sqrt{2N}}n-p}{t}\right) \end{array}$$

(20) $$\begin{array}{cc} n & =N\Psi \left(\frac{q+zm}{f}\right) \end{array}$$

1. m=0
  Since m=0, from (19), we know that:
  (21) $$v+\frac{b}{\sqrt{2N}}n-p\leq 0;$$
  and (20) becomes:
  (22) $$n=N\Psi \left(\frac{q}{f}\right).$$
  (a) n=0
    From (21) and (22), we obtain, respectively:
    (23) $$\begin{array}{cc} p & \geq v, \end{array}$$
    (24) $$\begin{array}{cc} q & \leq 0. \end{array}$$
  (b) 0<n<N
    Now:
    (25) $$n=N\frac{q}{f}$$
    and, again, from (21) and (22),
    (26) $$\begin{array}{cc} p-\frac{b\sqrt{N/2}}{f}q & \geq v, \end{array}$$
    (27) $$\begin{array}{cc} 0 & <q<f. \end{array}$$
  (c) n=N
    Once again, from (21) and (22):
    (28) $$\begin{array}{cc} p & \geq 2C, \end{array}$$
    (29) $$\begin{array}{cc} q & \geq f. \end{array}$$
2. 0<m<M
  Now:
  (30) $$0<v+\frac{b}{\sqrt{2N}}n-p<t,$$
  and:
  (31) $$m=\frac{M}{t}\left(v+\frac{b}{\sqrt{2N}}n-p\right).$$
  (a) n=0
    Equation (31) becomes:
    (32) $$m=\frac{M}{t}\left(v-p\right).$$
    From (30) and (20), we obtain, respectively,
    (33) $$\begin{array}{cc} v-t & <p<v, \end{array}$$
    (34) $$\begin{array}{cc} p-\frac{t}{Mz}q & \geq v. \end{array}$$
  (b) 0<n<N
    (35) $$\begin{array}{cc} m & =\frac{1}{zD}\left(v-p+\frac{b\sqrt{N/2}}{f}q\right), \end{array}$$
    (36) $$\begin{array}{cc} n & =\frac{N}{fD}\left(v-p+\frac{t}{Mz}q\right) \end{array}$$
    and:
    (37) $$\begin{array}{cc} 0 & <\frac{1}{D}\left(v-p+\frac{b\sqrt{N/2}}{f}q\right)<Mz \end{array}$$
    (38) $$\begin{array}{cc} 0 & <\frac{1}{D}\left(v-p+\frac{t}{Mz}q\right)<f. \end{array}$$
  (c) n=N
    (39) $$m=\frac{M}{t}\left(2C-p\right)$$
    and:
    (40) $$\begin{array}{cc} 2C-t & <p<2C, \end{array}$$
    (41) $$\begin{array}{cc} p-\frac{t}{Mz}q & \leq 2C-\frac{ft}{Mz}. \end{array}$$
3. m=M
  Now:
  (42) $$v+\frac{b}{\sqrt{2N}}n-p\geq t,$$
  and:
  (43) $$n=N\Psi \left(\frac{q+Mz}{f}\right).$$
  (a) n=0
    (44) $$\begin{array}{cc} p & \leq v-t, \end{array}$$
    (45) $$\begin{array}{cc} q & \leq -Mz \end{array}$$
  (b) 0<n<N
    (46) $$n=N\frac{q+Mz}{f}.$$
    and:
    (47) $$\begin{array}{cc} p-\frac{b\sqrt{N/2}}{f}q & \leq v-t+Mz\frac{b\sqrt{N/2}}{f}, \end{array}$$
    (48) $$\begin{array}{cc} -Mz & <q<f-Mz \end{array}$$
  (c) n=N
    (49) $$\begin{array}{cc} p & \leq 2C-t, \end{array}$$
    (50) $$\begin{array}{cc} q & \geq f-Mz \end{array}$$

Table 1 summarizes the conditions on *p* and *q* for each solution type that has been derived above. Let us denote by $R_{11}$ the feasibility region corresponding to the case in which m=n=0; by $R_{12}$, the region for the case m=0, 0<n<N; … and by $R_{33}$, the region for the case m=M, n=N. It is easy to check that, altogether, the nine regions cover the whole pq-plane (i.e., ${⋃}_{i,j=1,2,3}R_{ij}=R^{2}$). It is also clear that $R_{22}$ is bounded, whereas the remaining eight regions are unbounded. We further introduce the following notation for regions in the pq-plane: $\partial R_{ij}$ denotes the boundary of $R_{ij}$ and ${\bar{R}}_{ij}=R_{ij}\cup \;\partial R_{ij}$ the closure of $R_{ij}$ . With this notation, we have, for example:

(51) $$\begin{array}{cc} R_{22} & =\left\{\left(p,q\right):0<\frac{1}{D}\left(v-p+\frac{b\sqrt{N/2}}{f}q\right)<Mz,\;0<\frac{1}{D}\left(\;v-p+\frac{t}{Mz}q\right)<f\right\}, \end{array}$$

(52) $$\begin{array}{cc} \partial R_{22} & =\left\{\left(p,q\right):\frac{1}{D}\left(v-p+\frac{b\sqrt{N/2}}{f}q\right)=0,\;0\leq \frac{1}{D}\left(\;v-p+\frac{t}{Mz}q\right)\leq f\right\} \end{array}$$

(53) $$\begin{array}{c} \;⋃\;\left\{\left(p,q\right):\frac{1}{D}\left(v-p+\frac{b\sqrt{N/2}}{f}q\right)=Mz,\;0\leq \frac{1}{D}\left(\;v-p+\frac{t}{Mz}q\right)\leq f\right\} \end{array}$$

(54) $$\begin{array}{c} \;⋃\;\left\{\left(p,q\right):0\leq \frac{1}{D}\left(v-p+\frac{b\sqrt{N/2}}{f}q\right)\leq Mz,\;\frac{1}{D}\left(\;v-p+\frac{t}{Mz}q\right)=0\right\} \end{array}$$

(55) $$\begin{array}{c} \;⋃\;\left\{\left(p,q\right):0\leq \frac{1}{D}\left(v-p+\frac{b\sqrt{N/2}}{f}q\right)\leq Mz,\;\frac{1}{D}\left(\;v-p+\frac{t}{Mz}q\right)=f\right\}, \end{array}$$

(56) $$\begin{array}{cc} {\bar{R}}_{22} & =\left\{\left(p,q\right):0\leq \frac{1}{D}\left(v-p+\frac{b\sqrt{N/2}}{f}q\right)\leq Mz,\;0\leq \frac{1}{D}\left(\;v-p+\frac{t}{Mz}q\right)\leq f\right\}; \end{array}$$

that is, $\partial R_{22}$ is made up of four line segments, which correspond to the edges of a parallelogram; $R_{22}$ contains the interior points of the parallelogram; and ${\bar{R}}_{22}$ is the complete parallelogram (interior and edges).

The expressions for *m* and *n* in each region are summarized in Table 2.

It is easily seen that both *m* and *n*, as functions of *p* and *q*, are continuous; and therefore, so is $\Pi_{p}$, which is defined in (3). The expression in each region for the function $\Pi_{p}\left(p,q\right)$ is given in Table 3, where:

(57) $$G\left(p,q\right)\equiv \Pi_{p}\left(p,q\right){|}_{R_{22}}=\frac{p}{zD}\left(v-p+\frac{b\sqrt{N/2}}{f}q\right)-\frac{N}{fD^{2}}{\left(v-p+\frac{t}{Mz}q\right)}^{2}.$$

### 3.2. Maximization of the Provider’s Profit *Π*_p_(p,q)

Now, we study the maximum of $\Pi_{p}\left(p,q\right)$ in each region ${\bar{R}}_{ij}$, except for the case of the central one (${\bar{R}}_{22}$), which is addressed separately. Our purpose here is to determine a set of candidate points at which the global maximum of $\Pi_{p}\left(p,q\right)$, which we denote by $\pi^{*}$, is achieved.

Let us introduce the notation:

(58) $$\begin{array}{cc} \pi_{ij} & =\max_{\left(p,q\right)\in {\bar{R}}_{ij}}\Pi_{p}\left(p,q\right) \end{array}$$

(59) $$\begin{array}{cc} \Gamma_{ij} & =\left\{\left(p,q\right)\in {\bar{R}}_{ij}:\Pi_{p}\left(p,q\right)=\pi_{ij}\right\}. \end{array}$$

Now, by solving a simple optimization problem in each region, we can easily obtain:

(60) $$\begin{array}{cc} \pi_{11} & =0 \end{array}$$

(61) $$\begin{array}{cc} \Gamma_{11} & ={\bar{R}}_{11} \end{array}$$

(62) $$\begin{array}{cc} \pi_{12} & =0=\pi_{11} \end{array}$$

(63) $$\begin{array}{cc} \Gamma_{12} & =\left\{(p,0):p\geq v\right\}\subset \Gamma_{11} \end{array}$$

(64) $$\begin{array}{cc} \pi_{13} & =-Nf<0=\pi_{11} \end{array}$$

(65) $$\begin{array}{cc} \Gamma_{13} & ={\bar{R}}_{13} \end{array}$$

(66) $$\begin{array}{cc} \pi_{21} & =\left\{\begin{array}{cc} 0 & \mathrm{if}\;v\leq 0\\ \pi_{31} & \mathrm{if}\;0\leq t\leq v/2\\ M{\left(v/2\right)}^{2}/t & \mathrm{if}\;0<v/2\leq t \end{array}\right. \end{array}$$

(67) $$\begin{array}{cc} \Gamma_{21} & =\left\{\begin{array}{cc} \left\{(v,q):q\leq 0\right\}\subset \Gamma_{11} & \mathrm{if}\;v\leq 0\\ \Gamma_{31} & \mathrm{if}\;0\leq t\leq v/2\\ \left\{\left(v/2,q\right):q\leq -\frac{Mz}{t}\frac{v}{2}\right\} & \mathrm{if}\;0<v/2\leq t \end{array}\right. \end{array}$$

(68) $$\begin{array}{cc} \pi_{23} & =\left\{\begin{array}{cc} -Nf=\pi_{13} & \mathrm{if}\;C\leq 0\\ M\left(2C-A-t\right)=\pi_{33} & \mathrm{if}\;0\leq t\leq C\\ M(C^{2}/t-A) & \mathrm{if}\;0<C\leq t \end{array}\right. \end{array}$$

(69) $$\begin{array}{cc} \Gamma_{23} & =\left\{\begin{array}{cc} \left\{(2C,f)\right\}\subset \Gamma_{13} & \mathrm{if}\;C\leq 0\\ \Gamma_{33} & \mathrm{if}\;0\leq t\leq C\\ \left\{(C,f-\frac{Mz}{t}C)\right\} & \mathrm{if}\;0<C\leq t \end{array}\right. \end{array}$$

(70) $$\begin{array}{cc} \pi_{31} & =M(v-t) \end{array}$$

(71) $$\begin{array}{cc} \Gamma_{31} & =\left\{(v-t,q):q\leq -Mz\right\} \end{array}$$

(72) $$\begin{array}{cc} \pi_{32} & =\left\{\begin{array}{cc} M\left(v+AB^{2}-t\right)>\pi_{33} & \mathrm{if}\;B<1\\ \pi_{33} & \mathrm{if}\;B\geq 1 \end{array}\right. \end{array}$$

(73) $$\begin{array}{cc} \Gamma_{32} & =\left\{\begin{array}{cc} \left\{(v-t+2AB^{2},fB-Mz)\right\} & \mathrm{if}\;B<1\\ \Gamma_{33} & \mathrm{if}\;B\geq 1 \end{array}\right. \end{array}$$

(74) $$\begin{array}{cc} \pi_{33} & =M(2C-A-t) \end{array}$$

(75) $$\begin{array}{cc} \Gamma_{33} & =\left\{(2C-t,f-Mz)\right\} \end{array}$$

We observe that $\Gamma_{ij}⋂\partial R_{22}\neq ⌀,\;i,j=1,2,3$, that is the maximum of $\Pi_{p}\left(p,q\right)$ outside $R_{22}$ (i.e., in $R^{2}\setminus R_{22}$) is achieved, at least, in a point of the boundary of $R_{22}$; additionally, it may be achieved, as well, at other points outside ${\bar{R}}_{22}$. Now, we restrict ourselves to the case in which the maximum profit is positive ($\pi^{*}>0$), which is the case of practical interest, and provide the conditions under which this occurs. As will be seen, when $\pi^{*}>0$, the value $\pi^{*}$ is only achieved in ${\bar{R}}_{22}$.

Therefore, we can now focus our attention on the maximum in ${\bar{R}}_{22}$ since we know it will also be the global maximum. The following proposition gives the sufficient and necessary conditions for the maximum to be achieved at an interior point (i.e., in $R_{22}$). Otherwise, it will be achieved on the boundary, and its value and location are given in Equations (60)–(75).

**Proposition** **1.** *When the optimum profit is positive*, $\pi^{*}=\max_{\left(p,q\right)\in {\bar{R}}_{22}}G\left(p,q\right)>0$, *the optimum* $\pi^{*}$ *is achieved at a unique interior point to* ${\bar{R}}_{22}$, $\left(p_{\mathrm{int}},q_{\mathrm{int}}\right)\in R_{22}$, *iff* v>0 *and* $t>t_{0}$, *where:*

(76) $$t_{0}=\frac{v}{2}\max\left(1,B\right)+AB^{2}=\left\{\begin{array}{cc} BC & \mathrm{if}\;B\geq 1\\ v/2+AB^{2} & \mathrm{if}\;B\leq 1, \end{array}\right.$$

(77) $$\left(p_{\mathrm{int}},q_{\mathrm{int}}\right)=\left(\frac{v/2}{t-AB^{2}}t,\frac{v/2(fB-Mz)}{t-AB^{2}}\right)$$

*and:*

(78) $$\pi^{*}=\pi_{\mathrm{int}}\equiv G\left(p_{\mathrm{int}},q_{\mathrm{int}}\right)=\frac{{\left(v/2\right)}^{2}}{t-AB^{2}}M.$$

**Proof.**  Note that since ${\bar{R}}_{22}$ is a closed and bounded set and the function G(p,q) (defined in (57)) is continuous, the existence of a maximum value is guaranteed. Furthermore, the set ${\bar{R}}_{22}$ is convex.

To simplify the notation, the following change of variables is introduced:

(79) $$\begin{array}{cc} x & =\frac{1}{D}\left(v-p+\frac{b\sqrt{N/2}}{f}q\right) \end{array}$$

(80) $$\begin{array}{cc} y & =\frac{1}{D}\left(v-p+\frac{t}{Mz}q\right), \end{array}$$

so that

$$G\left(x,y\right)=\frac{1}{z}\left(v-\frac{t}{Mz}x+\frac{b\sqrt{N/2}}{f}y\right)x-\frac{N}{f}y^{2}$$

and:

$${\bar{R}}_{22}=\left\{\left(x,y\right):0\leq x\leq Mz,\;0\leq y\leq f\right\}.$$

It is easy to check that:

(81) $$\begin{array}{cc} \nabla G(x,y) & =\left[\frac{1}{z}\left(v-\frac{2t}{Mz}x+\frac{b\sqrt{N/2}}{f}y\right),\frac{b\sqrt{N/2}}{zf}x-\frac{2N}{f}y\right] \end{array}$$

(82) $$\begin{array}{cc} \frac{\partial^{2}G}{\partial x^{2}} & =-\frac{2t}{Mz^{2}} \end{array}$$

(83) $$\begin{array}{cc} |H_{G}\left(x,y\right)| & =\frac{\partial^{2}G}{\partial x^{2}}\frac{\partial^{2}G}{\partial y^{2}}-{\left(\frac{\partial^{2}G}{\partial x\partial y}\right)}^{2}=\frac{4N}{fMz^{2}}\left(t-AB^{2}\right). \end{array}$$

First, let us assume that $\left(p_{\mathrm{int}},q_{\mathrm{int}}\right)\in R_{22}$ is the unique maximum of G(p,q) in ${\bar{R}}_{22}$. Applying the transformation of (79) and (80), we obtain:

(84) $$\left(x_{\mathrm{int}},y_{\mathrm{int}}\right)=\left(\frac{Mz}{t-AB^{2}}\frac{v}{2},\frac{fB}{t-AB^{2}}\frac{v}{2}\right),$$

Since $\left(x_{\mathrm{int}},y_{\mathrm{int}}\right)$ is a maximum, we must have $\nabla G\left(x_{\mathrm{int}},y_{\mathrm{int}}\right)=\left[0,\;0\right]$, $\frac{\partial^{2}G}{\partial x^{2}}\left(x_{\mathrm{int}},y_{\mathrm{int}}\right)<0$ and $|H_{G}\left(x_{\mathrm{int}},y_{\mathrm{int}}\right)|>0$. From the latter, it follows that $t\geq AB^{2}$. If $t=AB^{2}$, then necessarily, v=0 and G(x,y) could be rewritten as:

(85) $$G\left(x,y\right)=-\frac{N}{f}{\left(y-\frac{fB}{Mz}x\right)}^{2}\leq 0$$

so that all points in the line y=fB/(Mz)x would yield a maximum $\pi^{*}=0$, which contradicts the assumption that the maximum is unique and that $\pi^{*}>0$. Thus, we have $t>AB^{2}$. Besides, for $\left(x_{\mathrm{int}},y_{\mathrm{int}}\right)$ to be in $R_{22}$, the following conditions must hold:

$$\begin{array}{cc} 0 & <\frac{Mz}{t-AB^{2}}\frac{v}{2}<Mz\\ 0 & <\frac{fB}{t-AB^{2}}\frac{v}{2}<f. \end{array}$$

The first of them implies that v>0 and $t>v/2+AB^{2}$, and the second one implies v>0 and $t>Bv/2+AB^{2}$. Clearly, these two conditions can be summarized as v>0 and $t>t_{0}$.

Now, we assume that v>0 and $t>t_{0}$. Under these assumptions, it is easily seen that $\left(x_{\mathrm{int}},y_{\mathrm{int}}\right)\in R_{22}$, and $\nabla G\left(x_{\mathrm{int}},y_{\mathrm{int}}\right)=\left[0,\;0\right]$. Furthermore, $\left(x_{\mathrm{int}},y_{\mathrm{int}}\right)$ is the only possible stationary point. Moreover, $\frac{\partial^{2}G}{\partial x^{2}}<0$ and $|H_{G}|>0$ for all $\left(x,y\right)\in {\bar{R}}_{22}$. Hence, G(x,y) is strictly concave, and $\left(x_{\mathrm{int}},y_{\mathrm{int}}\right)$ is the unique global maximum in ${\bar{R}}_{22}$. ☐

We now establish some results for the case when the conditions of Proposition 1 do not hold, and thus, the global maximum $\pi^{*}$ must be obtained by comparing the candidates $\pi_{ij}$ given in Equations (60)–(75). For most of these results, the proof is immediate, and it is not provided.

**Lemma** **2.** $\pi_{32}>\pi_{31}$.

**Lemma** **3.** $\pi_{32}\geq \pi_{33}$.

**Lemma** **4.** *If* C≥0, $\pi_{23}\geq \pi_{33}$.

**Lemma** **5.** *If* C≥0 *and* B≥1, $\pi_{23}\geq \pi_{32}$.

**Lemma** **6.** *If* B≥1 *and* $\pi_{23}>0$ *then* $\pi_{23}>\pi_{21}$ *(the condition* $\pi_{23}>0$ *is motivated by the fact that we are interested in the case in which* $\pi^{*}>0$*)*.

**Proof.**  We assume that v>0. Otherwise, $\pi_{21}=0$, and the result is trivial.

Now, the following three cases are considered separately: t≤v/2, v/2<t<C and: $C\leq t\leq t_{0}=BC$.

If t≤v/2, then $\pi_{21}=M\left(v-t\right)<M\left(v-t\right)+MA\left(2B-1\right)=\pi_{32}\leq \pi_{23}$, the last inequality being a consequence of Lemma 5.

If v/2<t<C, $\pi_{23}>\pi_{21}$ is equivalent to:

(86) $$x\left(t\right)\equiv t+\frac{{\left(v/2\right)}^{2}}{t}<2C-A.$$

Since $x^{\prime }\left(t\right)=1-{\left((v/2)/t\right)}^{2}>0$ when t>v/2, we have:

$$\begin{array}{ccc} x(t) & \leq x(C)=2C-\frac{1}{C}(C+\frac{v}{2})(C-\frac{v}{2}) & \\ & \leq 2C-(C-\frac{v}{2}) & \left(\mathrm{since}\;v>0\right)\\ & =2C-AB & \left(\mathrm{by}\;\mathrm{Lemma}\;1\right)\\ & \leq 2C-A & (\mathrm{since}\;B\geq 1). \end{array}$$

If $C\leq t\leq t_{0}=BC$, $\pi_{23}>\pi_{21}$ is equivalent to:

$$\frac{1}{A}\left(C^{2}-\frac{v^{2}}{4}\right)>t.$$

Indeed,

$$\frac{1}{A}\left(C^{2}-\frac{v^{2}}{4}\right)=\frac{1}{A}\left(C+\frac{v}{2}\right)\left(C-\frac{v}{2}\right)=\frac{1}{A}\left(C+\frac{v}{2}\right)AB>BC=t_{0}\geq t.$$

☐

**Lemma** **7.** If B≤1, $t\leq t_{0}$ and $\pi_{32}>0$, then $\pi_{32}>\pi_{21}$.

**Proof.**  We assume that v>0. Otherwise, $\pi_{21}=0$, and the result is trivial.

We consider separately the case when t≤v/2 and when $v/2<t\leq t_{0}$.

If t≤v/2, then $\pi_{21}=M\left(v-t\right)<M\left(v-t\right)+AB^{2}=\pi_{32}$.

If $v/2<t\leq t_{0}=v/2+AB^{2}$, then $\pi_{32}>\pi_{21}$ is equivalent to:

(87) $$x\left(t\right)<v+AB^{2},$$

where x(t) is defined in (86).

Since $x^{\prime }\left(t\right)>0$ when t>v/2, we have:

$$x\left(t\right)\leq x\left(t_{0}\right)=t_{0}+\frac{{\left(v/2\right)}^{2}}{t_{0}}<t_{0}+\frac{{\left(v/2\right)}^{2}}{v/2}=v+AB^{2},$$

where the second inequality follows from $t_{0}>v/2$. ☐

**Proposition** **2.** *Let* B≥1.

*If* C≤A/2, *then:*

$$\pi_{23}\leq 0\;\mathrm{for}\;\mathrm{all}\;\;t\geq 0.$$

*On the other hand, if* C>A/2, *let:*

(88) $$t_{c}\equiv \left\{\begin{array}{cc} 2C-A\leq C & \mathrm{if}\;C\leq A\\ C^{2}/A\geq C & \mathrm{if}\;C\geq A. \end{array}\right.$$

*Then:*

$$\begin{array}{cc} \pi_{23} & >0\;\mathrm{for}\;t\in [0,t_{c}),\;\mathrm{and}\\ \pi_{23} & \leq 0\;\mathrm{for}\;t\geq t_{c}. \end{array}$$

*Furthermore*, $t_{c}>t_{0}=BC\geq C$ *iff* v>0.

**Proof.**  The first part of the Proposition follows immediately by observing that, if C≥0, $\pi_{23}$ is a continuous and decreasing function of *t*.

We now proceed to proof the second part of the proposition.

If v>0, by applying Lemma 1, we have: C=v/2+AB>AB≥A. Hence, $t_{c}=A^{-1}C^{2}=A^{-1}\left(\frac{v}{2}+AB\right)C>A^{-1}ABC=BC.$

Now, assume that $t_{c}>BC$. Thus, from the definition of $t_{c}$, it follows that CA>0 and $C^{2}/A>BC$. The last inequality implies that C/A−B>0, and applying again Lemma 1, we obtain v/2>0, which completes the proof. ☐

Note that the condition C≤A/2 is equivalent to v≤−A(2B−1)≤−A, the last inequality being a consequence of the assumption B≥1.

**Proposition** **3.** *Let* B≤1.

*If* $v\leq -AB^{2}$, *then:*

$$\pi_{32}=M\left(v+AB^{2}-t\right)\leq 0\;\mathrm{for}\;\mathrm{all}\;\;t\geq 0.$$

*On the other hand, if* $v>-AB^{2}$, *let:*

(89) $$t_{c}^{\prime }\equiv v+AB^{2}>0.$$

*Then:*

$$\begin{array}{cc} \pi_{32} & >0\;\mathrm{for}\;t\in [0,t_{c}^{\prime }),\;\mathrm{and}\\ \pi_{32} & \leq 0\;\mathrm{for}\;t\geq t_{c}^{\prime }. \end{array}$$

*Furthermore*, $t_{c}^{\prime }>t_{0}=v/2+AB^{2}$ *iff* v>0.

**Proof.**  The first part of the proposition follows immediately by observing that $\pi_{32}$, as a function of *t*, is continuous and decreasing.

The second part follows by noting that: $t_{c}^{\prime }-t_{0}=v+AB^{2}-\left(v/2+AB^{2}\right)=v/2.$ ☐

Combining the results above, we obtain the following theorem that provides a full characterization of the global maximum $\pi^{*}$.

**Theorem** **1.**

B≥1
  - v≤−A(2B−1)<0
    $$\pi^{*}=0\;\mathrm{for}\;\mathrm{all}\;t\geq 0$$
  −A(2B−1)<v<−A(2B−2)
    $$\pi^{*}=\left\{\begin{array}{cc} M(2C-A-t) & \mathrm{if}\;0\leq t<t_{c}\leq C\\ 0 & \mathrm{if}\;t\geq t_{c} \end{array}\right.$$
    *where* $t_{c}$ *is defined in* (88)*.*
  −A(2B−2)≤v≤0
    $$\pi^{*}=\left\{\begin{array}{cc} M(2C-A-t) & \mathrm{if}\;0\leq t\leq C\\ M(C^{2}/t-A) & \mathrm{if}\;C<t<t_{c}\\ 0 & \mathrm{if}\;t\geq t_{c}. \end{array}\right.$$
  - v>0
    $$\pi^{*}=\left\{\begin{array}{cc} M(2C-A-t) & \mathrm{if}\;0\leq t\leq C\\ M(C^{2}/t-A) & \mathrm{if}\;C<t\leq t_{0}\\ M{\left(v/2\right)}^{2}/\left(t-AB^{2}\right) & \mathrm{if}\;t\geq t_{0} \end{array}\right.$$
    *where* $t_{0}$ *is defined in* (76).
B≤1
  - $v\leq -AB^{2}<0$
    $$\pi^{*}=0\;\mathrm{for}\;\mathrm{all}\;t\geq 0$$
  $-AB^{2}<v\leq 0$
    $$\pi^{*}=\left\{\begin{array}{cc} M\left(v-t\right)+MAB^{2} & \mathrm{if}\;0\leq t<t_{c}^{\prime }\\ 0 & \mathrm{if}\;t\geq t_{c}^{\prime } \end{array}\right.$$
    *where* $t_{c}^{\prime }$ *is defined in* (89).
  - v>0
    $$\pi^{*}=\left\{\begin{array}{cc} M\left(v-t\right)+NfB^{2} & \mathrm{if}\;0\leq t\leq t_{0}\\ M{\left(v/2\right)}^{2}/\left(t-AB^{2}\right) & \mathrm{if}\;t>t_{0} \end{array}\right.$$
    *where* $t_{0}$ *is defined in* (76).

Let $\left(p^{*},q^{*}\right)$ denote the pair of prices at which the maximum is attained (i.e., $\Pi_{p}\left(p^{*},q^{*}\right)=\pi^{*}$). We note that when $\pi^{*}$ is positive, it is equal to either $\pi_{\mathrm{int}}$, $\pi_{23}$ (with C>0), $\pi_{32}$ or $\pi_{33}$. In all of these cases, the maximum is attained at a single point. Therefore, if $\pi^{*}>0$, the pair $\left(p^{*},q^{*}\right)$ that yields the maximum profit is unique.

Table 4 and Table 5 show a summary of the solution to the profit maximization problem. When $\pi^{*}>0$, four different solution types have been identified, which are denoted as ‘mn’, ‘Mn’, ‘mN’ and ‘MN’, depending on the values of *m* and *n* at the maximum.

## 4. Results and Discussion

In this section, we discuss the numerical results for the model analysed in the previous section. We present the results separately for the case where B>1 and the results for the case where B<1. In both cases, we show the solutions to the profit maximization problem with parameter values b=1, z=0.1, M=40 and N=30. The value of *f* is set to f=1 for the case where B>1 and f=3 for the case where B<1. The results discussed are the values in the solution of the number of subscribers, *m*, the number of WSIPs that join the platform, *n*, the price charged to each subscriber for the service, *p*, the payment to each WSIP, q+zm, the provider profit, $\Pi_{p}$, and the mean value of the profit of a connected WSIP, $\Pi_{w}$. Throughout this section, the results are represented as a function of the cost factor, *t*, and the intrinsic utility, *v*.

### 4.1. Optimum Analysis for a Large Users’ Basin (B > *1*)

**Figure 2:**
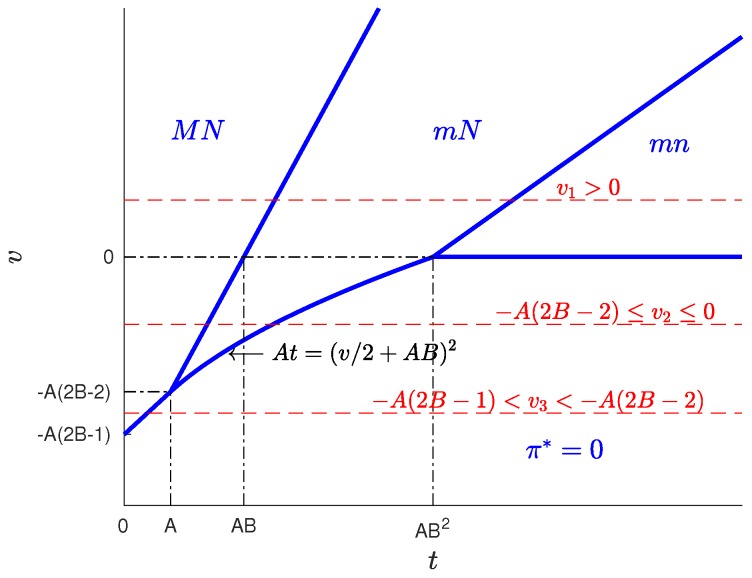
Regions of the solution types for B>1. M=40, N=30, f=1, b=1, z=0.1.

We first discuss the results for f=1, which gives a value of B>1. This implies that the number of users is large in relation to the number of WSNs. Specifically, this occurs when $M>\left(f/b\right)\sqrt{8N}$, i.e., when the number of users exceeds a certain threshold that depends on the number of WSIPs and parameters *f* and *b*. For this case, four solution types are possible, depending on the *t* and *v* values: MN, mN, mn and $\pi^{*}=0$. Figure 2 shows the regions in the tv-plane corresponding to each type of solution.

**Figure 3:**
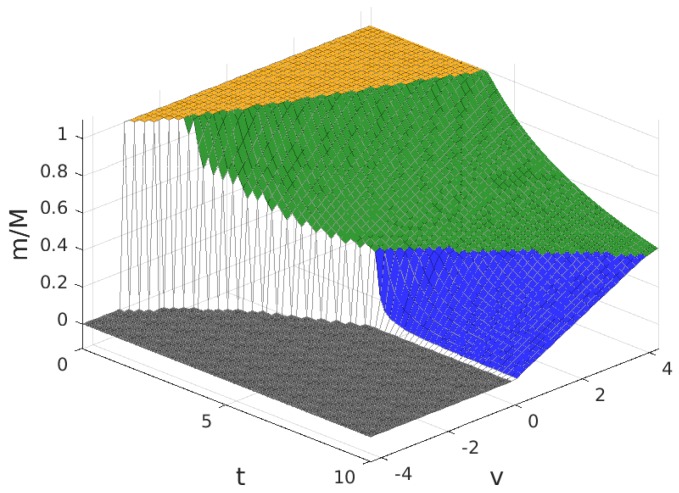
Fraction of users that subscribe, m/M, for B>1. Solution type: MN (yellow), mN (green), mn (blue) and $\pi^{*}=0$ (grey).

**Figure 4:**
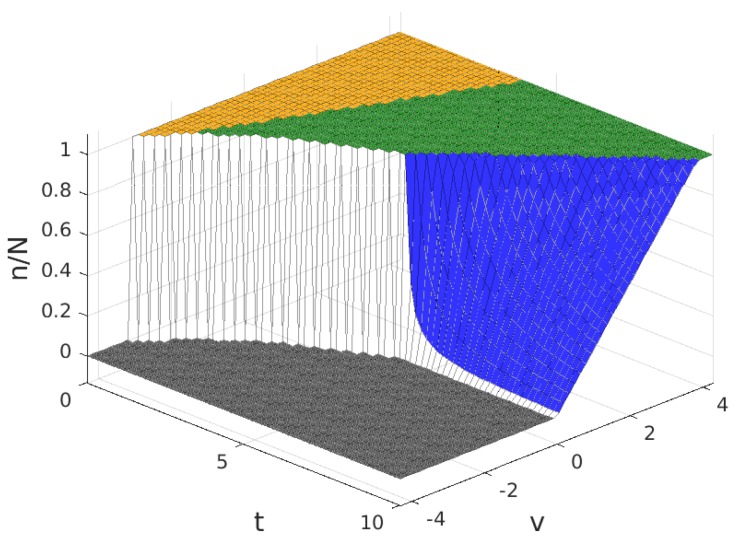
Fraction of WSNs that join the platform, n/N (equivalent to the payment to each Wireless Sensor Infrastructure Providers (WSIPs), (q+mz)/f and to the mean value of the profit of a connected WSIP, $\Pi_{w}/\left(f/2\right)$) for B>1.

**Figure 5:**
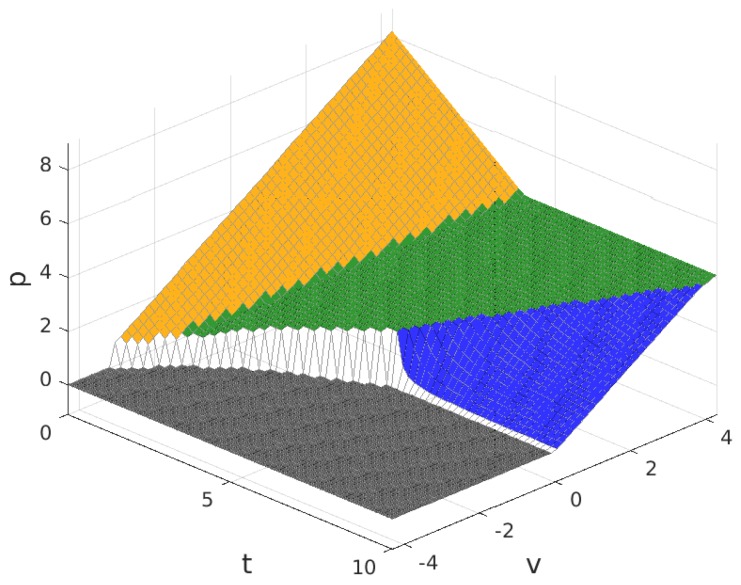
Price charged to each subscriber for the service, *p*, for B>1.

**Figure 6:**
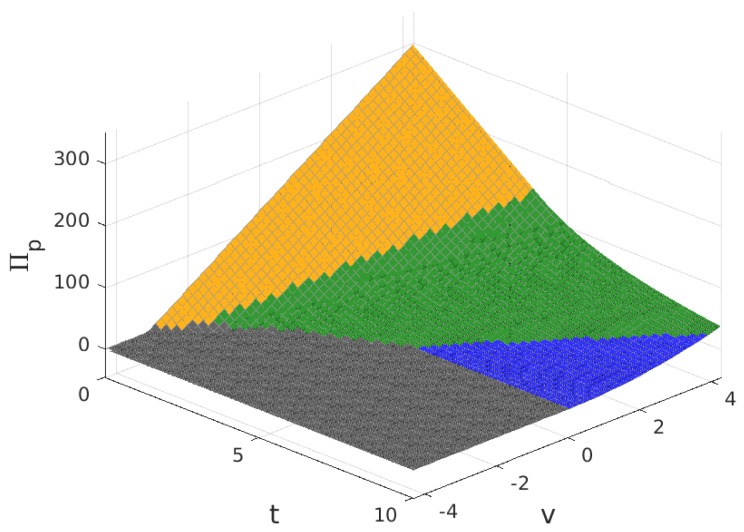
Provider profit, $\Pi_{p}$, for B>1.

The values for the solution are plotted in Figure 3, Figure 4, Figure 5 and Figure 6. Note that the values of q+zm and $\Pi_{w}$, in all of the solution types (see Table 5), are proportional to the value of n/N shown in Figure 4, so the plot in this figure can also be interpreted as (q+zm)/f and as $\Pi_{w}/\left(f/2\right)$. In these figures, the colour of the surface representing the solution parameter indicates the type of solution (yellow for MN, green for mN, blue for mn and grey for $\pi^{*}=0$).

**Figure 7:**
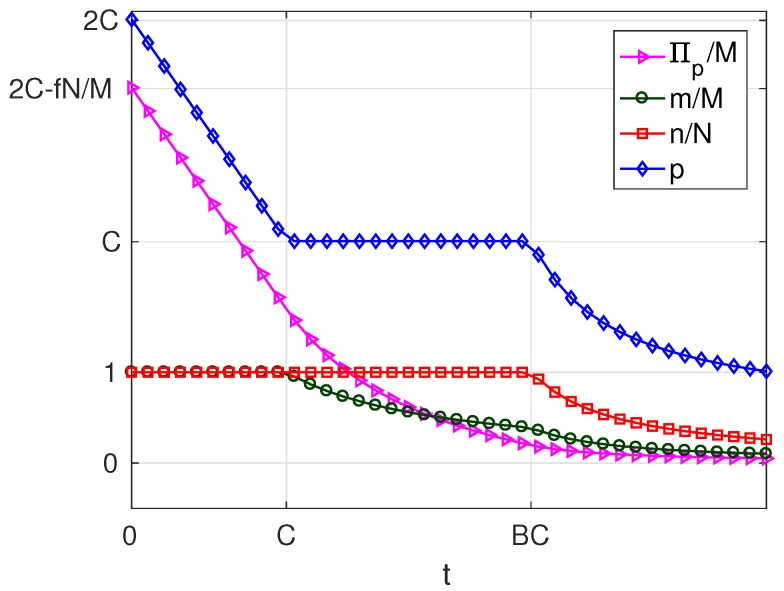
Solution for B>1 and $v=v_{1}$, ($v_{1}>0$).

**Figure 8:**
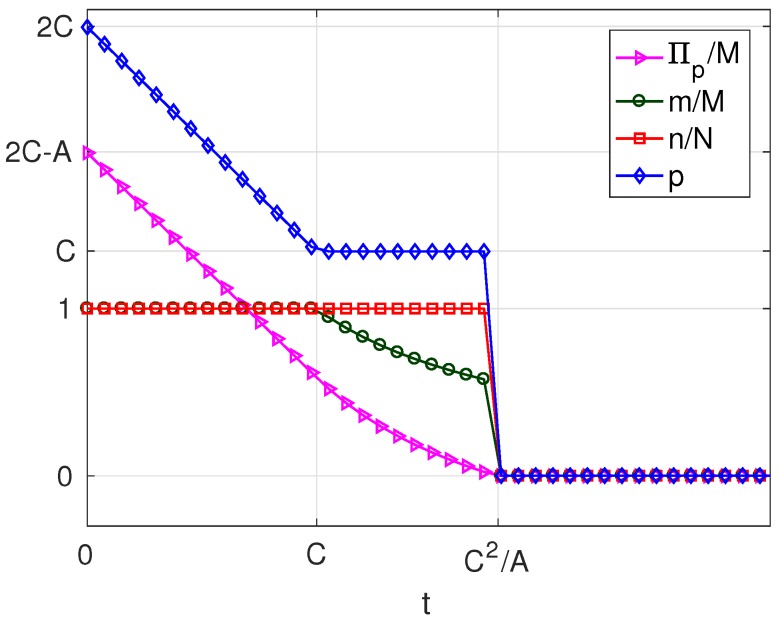
Solution for B>1 and $v=v_{2}$, ($-A\left(2B-2\right)\leq v_{2}\leq 0$).

**Figure 9:**
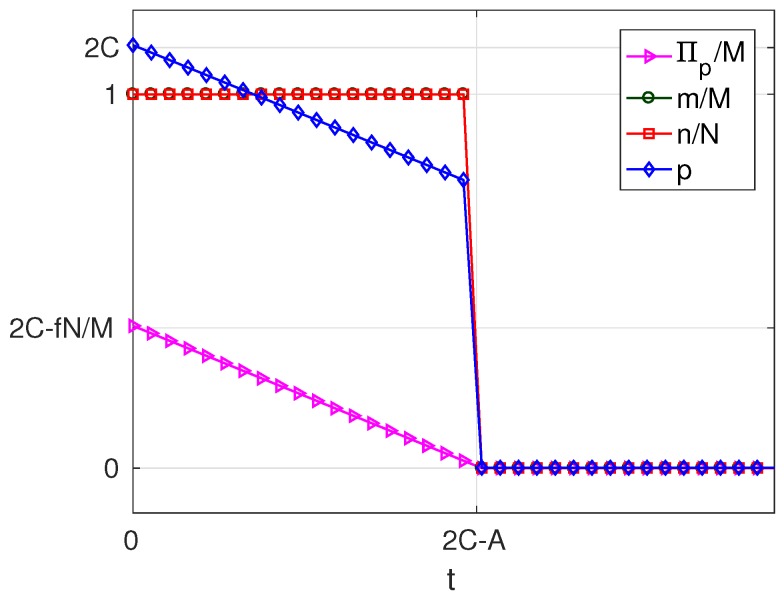
Solution for B>1 and $v=v_{3}$, ($-A\left(2B-1\right)<v_{3}<-A\left(2B-2\right)$).

As can be seen in Table 4, for B>1, the variation of the solution with *t* presents four different behaviours corresponding to four intervals of *v*. Of these, there are three intervals of *v* where a profitable business, i.e., $\pi^{*}>0$ is possible, which are: v>0 (in which solutions exist of types MN, mN and mn), −A(2B−2)≤v≤0 (in which solutions exist of types MN and mN) and −A(2B−1)<v<−A(2B−2) (in which only solutions of type MN exist). For a better understanding of the characteristics of the solution, for each of these intervals, we have set a value of the parameter *v* within the interval and represented the resulting solution as a function of the parameter *t*. The results are plotted in Figure 7, Figure 8 and Figure 9, corresponding to values of $v=v_{1}$, ($v_{1}>0$), $v=v_{2}$, ($-A\left(2B-2\right)\leq v_{2}\leq 0$) and $v=v_{3}$, ($-A\left(2B-1\right)<v_{3}<-A\left(2B-2\right)$), respectively. Each of these figures contains cross-sections of Figure 3, Figure 4, Figure 5 and Figure 6 for a fixed value of *v*. The locations of the three cross-sections have been represented in Figure 2.

1. If v>0, i.e., when subscribers receive a positive net value from accessing the platform irrespective of the amount of service received, we can state the following facts (Figure 7):
  - If t≤C, i.e., if the user costs are small compared with a quantity that increases with the number of WSIPs *N* and the strength of the externality *b*,
    – in the optimum, all users subscribe (m=M) and all WSIPs connect (n=N);
    – for t=0, the price and the server provider’s profit are maximum (p=2C and $\Pi_{p}=pM-fN=2CM-fN$);
    – as *t* increases, which means higher costs borne by the users, the platform chooses a lower *p* in order to compensate for the increase in *t*; and it succeeds in keeping m=M, but $\Pi_{p}$ decreases.
  - When C≤t≤BC, i.e., the user cost is maintained at an intermediate value,
    – the platform can no longer avoid that *m* decreases, so that it has no incentive to lower *p*, and *p* remains constant and equal to *C*;
    – $q^{B}+mz$ is maintained constant (by raising $q^{B}$), so that all WSIPs remain connected (n=N) and their profit $\Pi_{w}$ unaltered;
    – the decrease in *m* causes that service provider’s profit $\Pi_{p}$ decreases.
  - As *t* increases beyond BC, i.e., a quantity that increases almost linearly with *M* and $b^{2}$,
    – the platform chooses a lower price *p* and a lower *q* to try to compensate for the increase in *t*, but it cannot avoid that both *m* and *n* decrease asymptotically to zero;
    – the decrease in *n* causes both q+mz and $\Pi_{w}$ to decrease;
    – the decreases in *m* and in *n* cause $\Pi_{p}$ to decrease.
  The above facts show that, if v>0, there is a first user cost ceiling *C*, below which the take-ups *m* and *n* are maximum and above which *m* decreases while *n* is still maximum, and a second user cost ceiling BC above which both *m* and *n* decrease. Note that high values for *C* can be achieved in scenarios with a high availability of WSIPs and with a strong externality *b*.
2. If −A(2B−2)≤v≤0, i.e., when subscribers do not receive a positive net value from accessing the platform, but they pay a network access fee, which is higher than the value from accessing the platform, and *v* is below the threshold A(2B−2), we can state the following facts (Figure 8):
  - If t≤C, the solution has the same characteristics as in the case of B>1 and t≤C.
  - When $C\leq t\leq C^{2}/A$, the solution has the same characteristics as in the case of B>1 and C≤t≤BC, but now, the ceiling of this interval is minor ($C^{2}/A<BC$).
  - As *t* increases beyond $C^{2}/A$, *m* and *n* drop sharply to zero, which means that in this scenario, the mn-type solution is no longer possible, but instead, it passes directly from solution type mN to solution type $\pi^{*}=0$.
  The above facts show that, if −A(2B−2)≤v≤0, there is a user cost ceiling *C*, below which the take-ups *m* and *n* are maximum. Beyond this cost ceiling and up to $C^{2}/A$, the take-ups decrease, and above $C^{2}/A$, all users unsubscribe.
3. If −A(2B−1)<v<−A(2B−2), i.e., when the value of *v* is higher than in the previous case, but below the threshold A(2B−1), we can state that (Figure 9):
  - The only possible solution other than $\pi^{*}=0$ is of type MN, and it exists only if t≤2C−A.
  - When t≤2C−A, the solution has the same characteristics as in the previous case for t≤C.

Of the different regions of results shown for the case B>1, those that represent a more feasible business situation are those in which all WSNs join the platform and a fraction of the users subscribe. This result occurs when v>0 and C≤t≤BC (Figure 7) and when −A(2B−2)≤v≤0 and $C\leq t\leq C^{2}/A$ (Figure 8). Both regions have in common that the user cost is maintained at an intermediate value, and we have shown that they exist when the number of users exceeds a certain threshold that depends on the number of WSIPs and parameters *f* and *b*, that is, when the number of users is high enough in relation to the number of WSNs.

### 4.2. Optimum Analysis for a Small Users’ Basin (B < *1*)

Here, we discuss the results for f=3, which gives a value of B<1. This implies that $M<\left(f/b\right)\sqrt{8N}$, i.e., that the number of subscribers is under a threshold that depends on the number of WSIPs and parameters *f* and *b*. Note that this occurs if the number of users is low in relation to the number of WSNs, which may correspond to an unrealistic situation. However, for completeness, we present the results for B<1, and we show that in most regions of the vt-plane, they show some parallelism with those for B>1.

**Figure 10:**
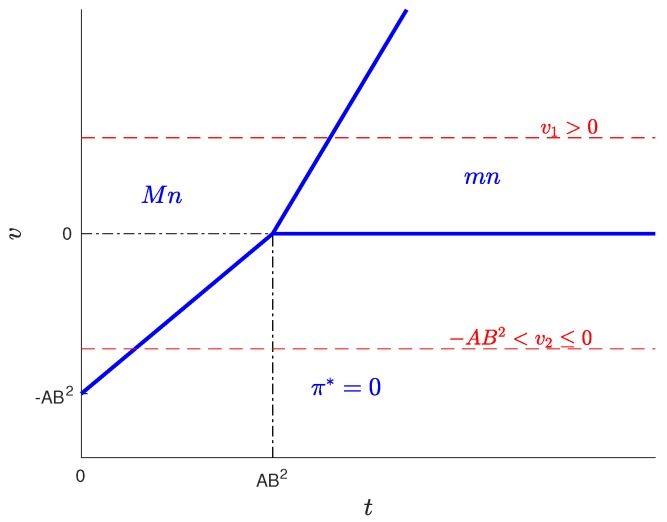
Regions of the solution types for B<1. M=40, N=30, f=3, b=1, z=0.1.

For this case, three solution types are possible, depending on the *t* and *v* values: Mn, mn and $\pi^{*}=0$. Figure 10 shows the regions of *t* and *v* corresponding to each type of solution.

**Figure 11:**
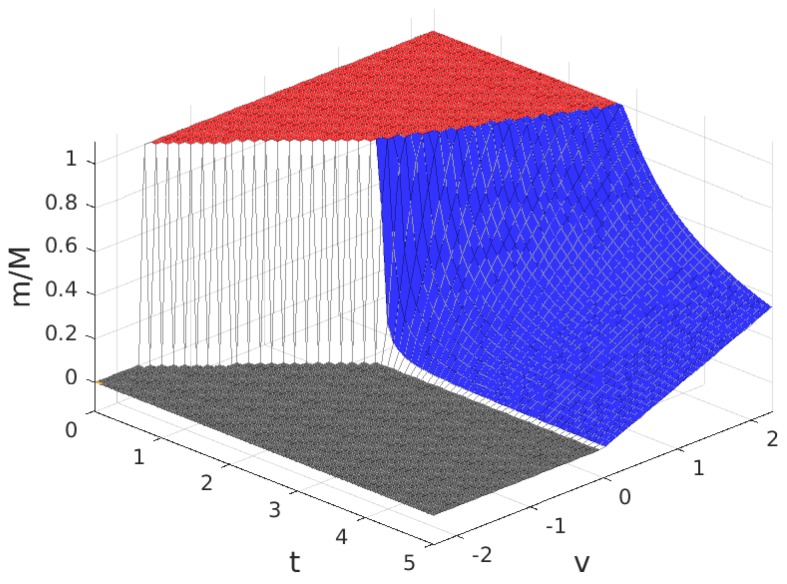
Fraction of users that subscribe, m/M, for B<1. Solution type: MN (red), mn (blue) and $\pi^{*}=0$ (grey).

**Figure 12:**
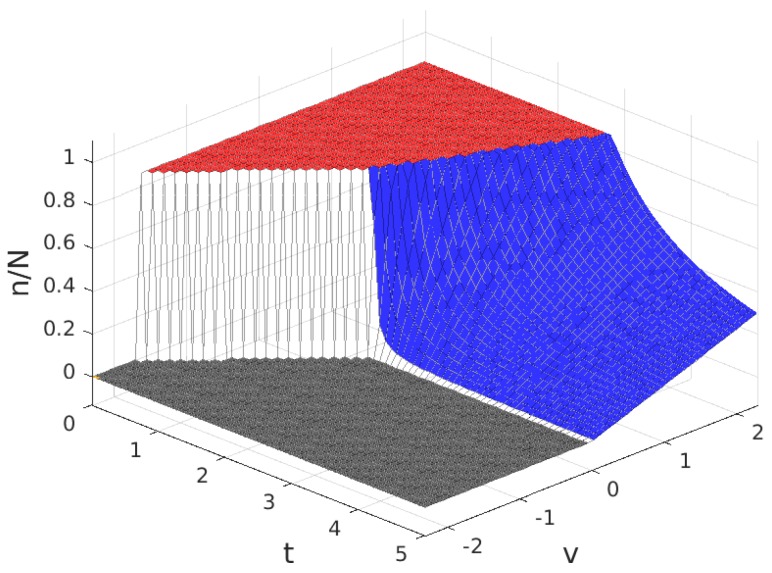
Fraction of WSNs that join the platform, n/N (equivalent to the payment to each WSIP, (q+mz)/f and to the mean value of the profit of a connected WSIP, $\Pi_{w}/\left(f/2\right)$) for B<1.

**Figure 13:**
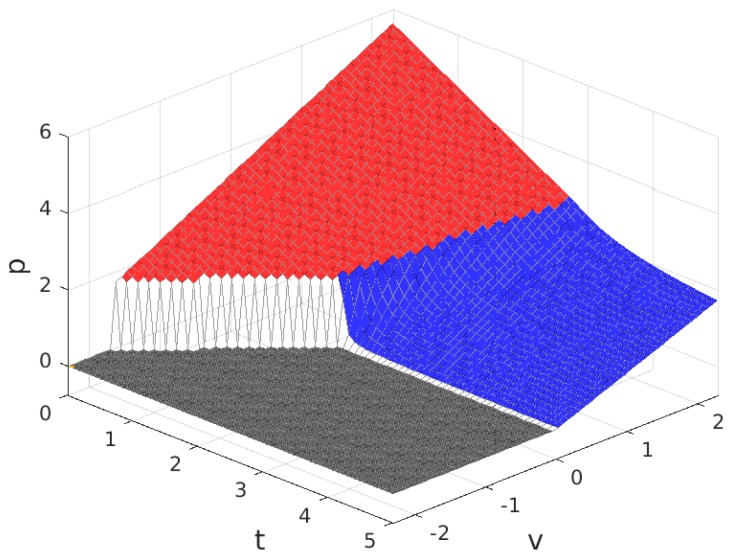
Price charged to each subscriber for the service, *p*, for B<1.

**Figure 14:**
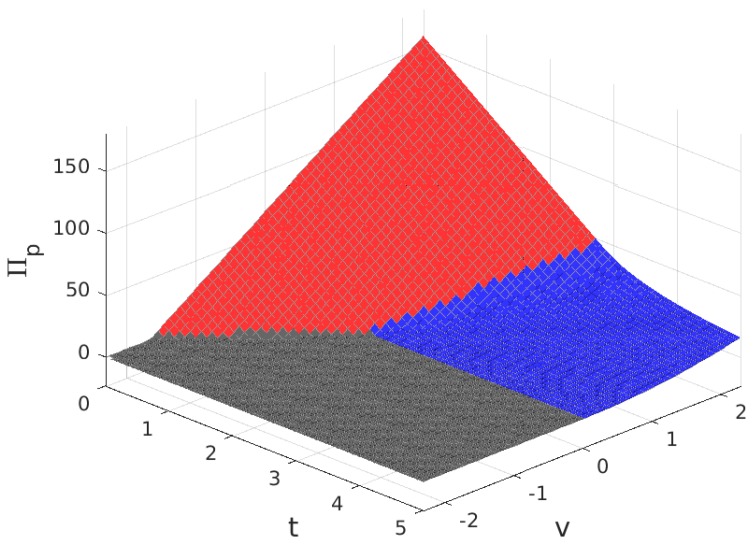
Provider profit, $\Pi_{p}$, for B<1.

The values for the solution are plotted in Figure 11, Figure 12, Figure 13 and Figure 14. As before, parameters q+zm and $\Pi_{w}$ are proportional to the value of n/N and can be derived from Figure 12, and the colour of the surface representing the solution parameter indicates the type of solution (red for Mn, blue for mn and grey for $\pi^{*}=0$).

**Figure 15:**
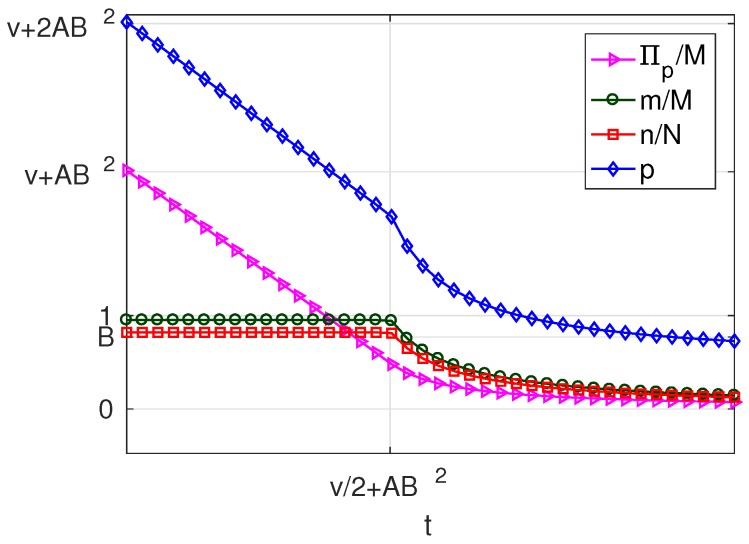
Solution for B<1 and $v=v_{1}$, ($v_{1}>0$).

**Figure 16:**
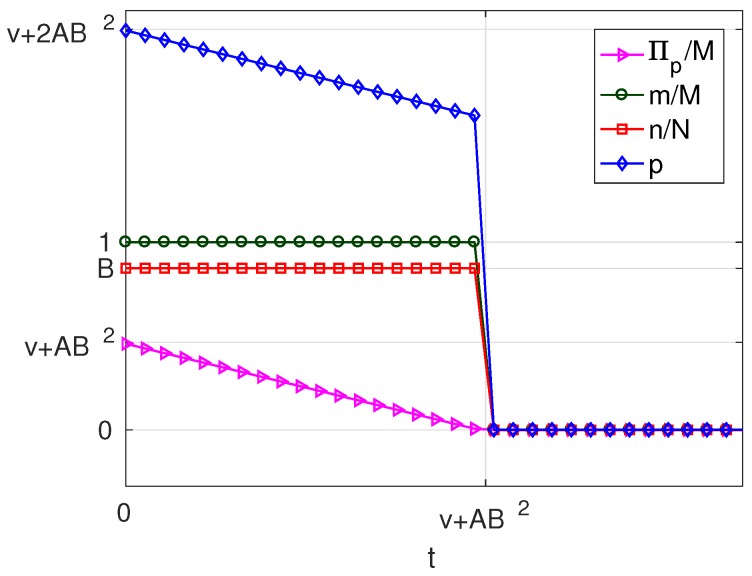
Solution for B<1 and $v=v_{2}$, ($-AB^{2}<v_{2}\leq 0$).

As can be seen in Table 4, for B<1, the variation of the solution with *t* presents three different behaviours corresponding to three intervals of *v*. Of these, there are two intervals of *v* in which solutions different from $\pi^{*}=0$ exist, which are: v>0 (in which solutions exist of types Mn and mn) and $-A^{2}B\leq v\leq 0$ (in which only solutions of type Mn exist). For each of these intervals, we have set a value of the parameter *v* within the interval and represented the resulting solution as a function of the parameter *t*. The results are plotted in Figure 15 and Figure 16, corresponding to values of $v=v_{1}$, ($v_{1}>0$) and $v=v_{2}$, ($-AB^{2}\leq v_{2}\leq 0$), respectively. Each of these figures contains cross-sections of Figure 11, Figure 12, Figure 13 and Figure 14 for a fixed value of *v*. The locations of the two cross-sections have been represented in Figure 10.

1. If v>0, i.e., when subscribers receive a positive net value from accessing the platform, we can state the following facts (Figure 15):
  - If $t\leq v/2+AB^{2}=v/2+Mb^{2}/8f$, i.e., if the user costs are small compared with a quantity that increases with *v*, the number of providers *M* and the strength of the cross externality *b*,
    – in the optimum, all users subscribe (m=M) and a fraction *B* of WSIPs connect (n=BN);
    – for t=0, the price and the server provider’s profit are maximum ($p=v+AB^{2}$ and $\Pi_{p}=vM+2AB^{2}M$);
    – as *t* increases, the platform reduces *p* in order to compensate for the increase in *t*; and it succeeds in keeping m=M, but $\Pi_{p}$ decreases.
  - When $t>v/2+AB^{2}$,
    – the platform chooses a lower price *p* and a lower *q* to try to compensate for the increase in *t*, but it cannot avoid that both *m* and *n* decrease asymptotically to zero;
    – the decrease in *n* causes both q+mz and $\Pi_{w}$ to decrease;
    – the decrease in *m* and in *n* causes $\Pi_{p}$ to decrease.
  The above facts show that, if v>0, there is a user cost ceiling $v/2+AB^{2}=v/2+Mb^{2}/8f$, below which the take-up *m* is maximum and *n* is maintained at a constant value BN. Beyond this cost ceiling, the take-ups decrease.
2. If $-AB^{2}\leq v\leq 0$, i.e., when users pay a positive net cost from accessing the platform (v<0), and this net cost is below the threshold $AB^{2}=Mb^{2}/8f$, we can state the following facts (Figure 16):
  - If $t\leq v+AB^{2}$, the solution has the same characteristics as in the previous case for $t\leq v/2+AB^{2}$.
  - As *t* increases beyond a threshold given by $v/2+AB^{2}$, *m* and *n* drop sharply to zero, which means that in this scenario, the mn-type solution is no longer possible, but instead, it passes directly from type Mn to type $\pi^{*}=0$.
  The above facts show that, if $-AB^{2}\leq v\leq 0$, there is a user cost ceiling $v+AB^{2}=v+Mb^{2}/8f$, below which the take-up *m* is maximum and *n* is maintained at a constant value BN. Beyond this cost ceiling, all users unsubscribe.

We have seen that with B<1, the most interesting solutions from the point of view of business viability do not exist. Now, the solutions go directly from the region in which all of the users subscribe to the region in which the number of subscribers and the number of joined WSNs decreases with *t*, without going through the intermediate region in which the number of joined WSNs remained constant. Furthermore, now, in no case do all WSNs join as happened with B>1 for small values of *v*, but now, only a fraction of the WSNs do.

## 5. Conclusions

A business model is analysed for a service platform that intermediates between WSNs and users. A payment method has been proposed and studied through the analytic solution of the profit maximization problem. The behaviour of the model for typical parameter settings has been described. The analysis reveals that the service provider maximizes its profit by incentivizing all users and all WSIPs to join the platform. This statement is true not only when the number of users is high, but also when it is moderate, provided that the costs that the users bear do not trespass a cost ceiling. This cost ceiling depends on the number of WSIPs, on the value of the intrinsic value of the service and on the externality that the WSIPs has on the user utility. When the users bear high costs or when the service in not valuable enough, maximum profit is achieved at the expense of lower users and WSIP take-ups.

## Figures and Tables

**Table 1:** Feasibility region for each solution type of (m,n).

	*n* = 0	0 < *n* < *N*	*n* = *N*
m=0	p≥v	$p-\frac{b\sqrt{N/2}}{f}q\geq v$	p≥2C
	q≤0	0<q<f	q≥f
0<m<M	v−t<p<v	$0<\frac{1}{D}\left(v-p+\frac{b\sqrt{N/2}}{f}q\right)<Mz$	2C−t<p<2C
	$p-\frac{t}{Mz}q\geq v$	$0<\frac{1}{D}\left(v-p+\frac{t}{Mz}q\right)<f$	$p-\frac{t}{Mz}q\leq 2C-\frac{ft}{Mz}$
m=M	p≤v−t	$p-\frac{b\sqrt{N/2}}{f}\left(q+Mz\right)\leq v-t$	p≤2C−t
	q+Mz≤0	0<q+Mz<f	q+Mz≥f

**Table 2:** Expressions for *m* and *n* in each region.

	n=0	0<n<N	n=N
m=0	m=0	m=0	m=0
	n=0	$n=N\frac{q}{f}$	n=N
0<m<M	$m=\frac{M}{t}\left(v-p\right)$	$m=\frac{1}{z}\frac{v-p+\frac{b\sqrt{N/2}}{f}q}{D}$	$m=\frac{M}{t}\left(2C-p\right)$
	n=0	$n=\frac{N}{f}\frac{v-p+\frac{t}{Mz}q}{D}$	n=N
m=M	m=M	m=M	m=M
	n=0	$n=N\frac{q+Mz}{f}$	n=N

**Table 3:** Expression for $\Pi_{p}\left(p,q\right)$ in each region.

	n=0	0<n<N	n=N
m=0	0	$-\frac{N}{f}q^{2}$	−Nf
0<m<M	$\frac{M}{t}\left(v-p\right)p$	G(p,q)	$\frac{M}{t}\left(2C-p\right)\left(p-Nz\right)-Nq$
m=M	Mp	$Mp-\frac{N}{f}{\left(q+Mz\right)}^{2}$	Mp−(q+Mz)N

**Table 4:** Summary of the solution to profit maximization problem.

*B*	*v*	*t*	Type of Solution
B≤1	$v\leq -AB^{2}$	t≥0	$\pi^{*}=0$
	$-AB^{2}<v\leq 0$	$0\leq t<v+AB^{2}$	Mn
		$t\geq v+AB^{2}$	$\pi^{*}=0$
	v>0	$0\leq t\leq v/2+AB^{2}$	Mn
		$t>v/2+AB^{2}$	mn
B≥1	v≤−A(2B−1)	t≥0	$\pi^{*}=0$
	−A(2B−1)<v<−A(2B−2)	0≤t<2C−A	MN
		t≥2C−A	$\pi^{*}=0$
	−A(2B−2)≤v≤0	0≤t≤C	MN
		$C<t<C^{2}/A$	mN
		$t\geq C^{2}/A$	$\pi^{*}=0$
	v>0	0≤t≤C	MN
		C<t≤BC	mN
		t>BC	mn

**Table 5:** Types of solution when the maximum profit is positive.

Type	*m*	*n*	$p^{*}$	$q^{*}+\mathrm{mz}$	$\pi^{*}$	$\Pi_{w}$
mn	$\frac{v/2}{t-AB^{2}}M$	$\frac{v/2}{t-AB^{2}}BN$	$\frac{v/2}{t-AB^{2}}t$	$\frac{n}{N}f$	$\frac{{\left(v/2\right)}^{2}}{t-AB^{2}}M$	$\frac{n}{N}\frac{f}{2}$
mN	$\frac{C}{t}M$	*N*	*C*	$\frac{n}{N}f$	$M(C^{2}/t-A)$	$\frac{n}{N}\frac{f}{2}$
Mn	*M*	BN	$v-t+2AB^{2}$	$\frac{n}{N}f$	$M(v-t+AB^{2})$	$\frac{n}{N}\frac{f}{2}$
MN	*M*	*N*	2C−t	$\frac{n}{N}f$	M(2C−t−A)	$\frac{n}{N}\frac{f}{2}$

## Abbreviations

The following abbreviations are used in this manuscript:

WSN	Wireless Sensor Network
IoT	Internet of Things
WSIP	Wireless Sensor Infrastructure Provider
